# Olanzapine-Induced Hyperphagia and Weight Gain Associate with Orexigenic Hypothalamic Neuropeptide Signaling without Concomitant AMPK Phosphorylation

**DOI:** 10.1371/journal.pone.0020571

**Published:** 2011-06-13

**Authors:** Johan Fernø, Luis Varela, Silje Skrede, María Jesús Vázquez, Rubén Nogueiras, Carlos Diéguez, Antonio Vidal-Puig, Vidar M. Steen, Miguel López

**Affiliations:** 1 Dr. Einar Martens' Research Group for Biological Psychiatry, Department of Clinical Medicine, University of Bergen, Bergen, Norway; 2 Center for Medical Genetics and Molecular Medicine, Haukeland University Hospital, Bergen, Norway; 3 Department of Physiology, School of Medicine, University of Santiago de Compostela-Instituto de Investigación Sanitaria (IDIS), Santiago de Compostela, Spain; 4 CIBER Fisiopatología de la Obesidad y Nutrición (CIBERobn), Santiago de Compostela, Spain; 5 Institute of Metabolic Science, Metabolic Research Laboratories, University of Cambridge, Addenbrooke's Hospital, Cambridge, United Kingdom; Sapienza University of Rome, Italy

## Abstract

The success of antipsychotic drug treatment in patients with schizophrenia is limited by the propensity of these drugs to induce hyperphagia, weight gain and other metabolic disturbances, particularly evident for olanzapine and clozapine. However, the molecular mechanisms involved in antipsychotic-induced hyperphagia remain unclear. Here, we investigate the effect of olanzapine administration on the regulation of hypothalamic mechanisms controlling food intake, namely neuropeptide expression and AMP-activated protein kinase (AMPK) phosphorylation in rats. Our results show that subchronic exposure to olanzapine upregulates neuropeptide Y (NPY) and agouti related protein (AgRP) and downregulates proopiomelanocortin (POMC) in the arcuate nucleus of the hypothalamus (ARC). This effect was evident both in rats fed *ad libitum* and in pair-fed rats. Of note, despite weight gain and increased expression of orexigenic neuropeptides, subchronic administration of olanzapine decreased AMPK phosphorylation levels. This reduction in AMPK was not observed after acute administration of either olanzapine or clozapine. Overall, our data suggest that olanzapine-induced hyperphagia is mediated through appropriate changes in hypothalamic neuropeptides, and that this effect does not require concomitant AMPK activation. Our data shed new light on the hypothalamic mechanism underlying antipsychotic-induced hyperphagia and weight gain, and provide the basis for alternative targets to control energy balance.

## Introduction

The successful use of antipsychotic drugs such as clozapine and olanzapine in the treatment of schizophrenia is hampered by their unwanted obesogenic effect and associated metabolic side effects [1], [2]. It is clear that in a medium to long-term perspective, metabolic dysregulation predisposes to cardiovascular disease (CVD) and premature death [3], but even in a shorter perspective, weight gain may reduce treatment compliance, increasing the risk of relapse of psychosis [4]. The underlying mechanisms of antipsychotic-induced weight gain are incompletely understood; however, their elucidation may identify alternative targetable pathways controlling energy balance.

Current evidence indicates that antipsychotic-induced weight gain and lipid disturbances may be explained by the antipsychotics' hyperphagic effects, linked to lack of satiation as observed in patients and in animal models [5], [6], [7], [8], [9]. The molecular events involved in antipsychotic-induced hyperphagia remain unclear, but the propensity of the different antipsychotics to increase food intake and weight gain is correlated with particular patterns of affinity for serotonergic, histaminergic and muscarinic receptors in the central nervous system (CNS) [10]. In particular, antagonism at serotonin 5HT2C and histamine H1 receptors in the hypothalamus seems to be relevant (for review; see [11]). As a crucial mediator in the control of energy intake and expenditure, the hypothalamus integrates a wide array of afferent signals, including hormones such as leptin, ghrelin and insulin, by modifying the expression of specific neuromodulators including orexigenic and anorexigenic neuropeptides. These include the orexigenic neuropeptide Y (NPY) and agouti-related peptide (AgRP), and the anorexigenic neuropeptide precursors proopiomelanocortin (POMC) and cocaine and amphetamine-regulated transcript (CART) [12], [13], [14], [15], [16], [17]. The hypothalamus is organized in anatomically discrete neuronal clusters known as nuclei, with the arcuate nucleus (ARC) considered the “master hypothalamic centre” for feeding control [16], [17]. The effect of antipsychotic drugs on the expression of appetite-regulating hypothalamic neuropeptides has been investigated in rodent models, but with equivocal results. Hypothalamic expression of NPY was increased by clozapine [18] but decreased by olanzapine [19] although neither of these studies reported effects on food intake or weight gain. On the other hand, in other studies monitoring antipsychotic-induced hyperphagia and weight gain, no transcriptional changes of hypothalamic neuropeptides were found [20], [21].

Recent investigations have also linked antipsychotic drug treatment to alterations in hypothalamic lipid metabolism. In an acute study on mice, it was proposed that H1 receptor-mediated activation of hypothalamic AMP-activated protein kinase (AMPK) represents an important mechanism of action for antipsychotic-induced hyperphagia [22]. AMPK, a sensor of energy homeostasis at the cellular level, integrates metabolic signals and regulates energy balance via modulation of hypothalamic fatty acid metabolism within the hypothalamus [15], [23], [24], [25], [26]. At the molecular level, AMPK phosphorylation (activation) in the hypothalamus leads to phosphorylation (inhibition) of acetyl-CoA carboxylase (ACC), thus reducing the flux of substrates through the fatty acid biosynthesis pathway and, most importantly, lowering levels of malonyl-CoA with resultant orexigenic effects [13], [27].

Despite the fact that rodent models of antipsychotic-induced metabolic disturbances do not consistently recapitulate the human clinical phenotype, they are still extensively used preclinically (for review; see [28]). In rats, olanzapine frequently mimics the weight-promoting effect observed in patients, whereas comparable effects of clozapine are typically not reproduced in rodents [29], [30]. Furthermore, the olanzapine-induced hyperphagia and weight gain commonly observed in female rats and mice [29], [31], [32], [33], [34], [35], [36], [37] are less robustly demonstrated in male littermates [29], [38], [39]. To study potential molecular mechanisms involved in antipsychotic-induced hyperphagia, we therefore chose to use female Sprague-Dawley rats subchronically treated with olanzapine. In addition, acute effects of both olanzapine and clozapine were investigated in female rats. We demonstrate that subchronic exposure to olanzapine upregulates the orexigenic neuropeptides NPY and AgRP and downregulates the anorexigenic neuropeptide precursor POMC in the ARC. This effect was evident in both *ad libitum* and pair-fed female rats. Notably, despite weight gain and increases in orexigenic neuropeptides, AMPK phosphorylation levels were decreased by olanzapine in *ad libitum*-fed female rats, suggesting that olanzapine-induced orexigenic effects and neuropeptide expression changes in the subchronic setting may be regulated without concomitant AMPK activation.

## Results

### Effect of acute olanzapine administration on hypothalamic AMPK phosphorylation

**Figure 1:**
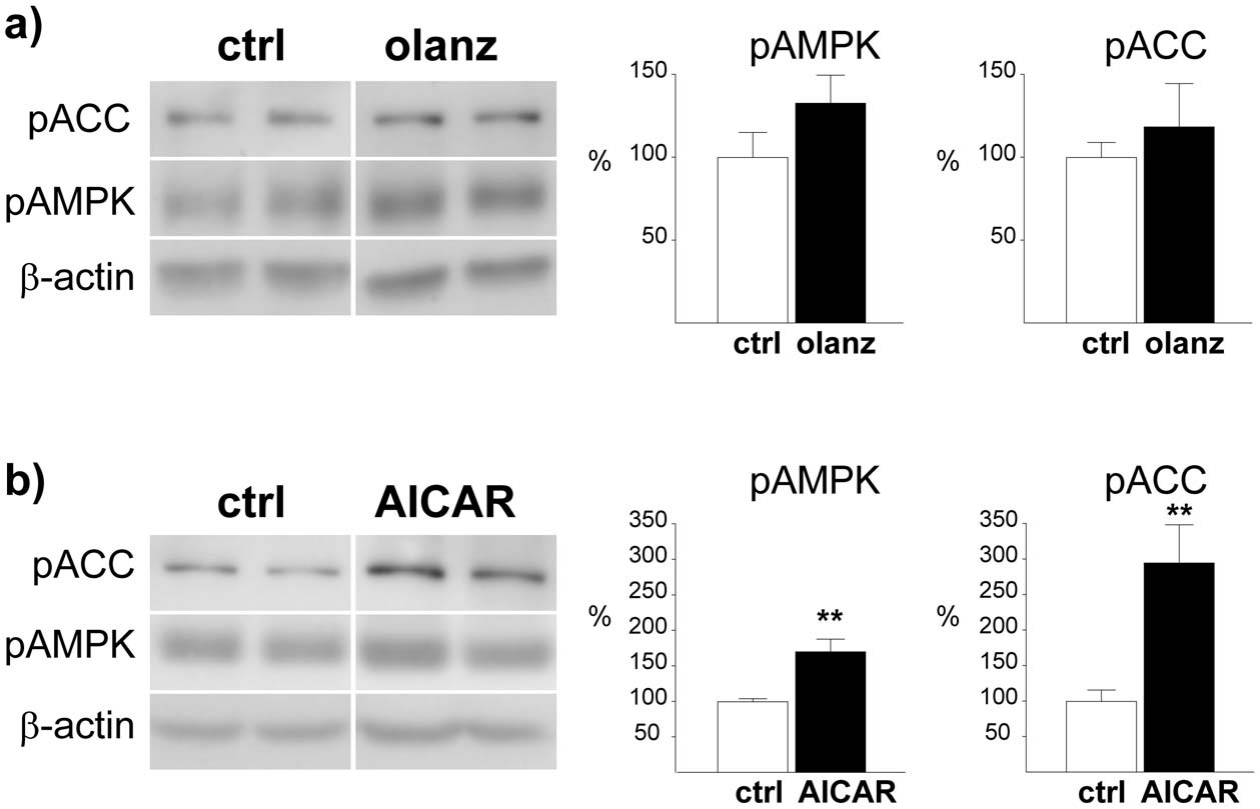
Effect of ICV olanzapine and AICAR administration on phosphorylation of hypothalamic AMPK and ACC. Western blot analysis of hypothalamic pAMPK and pACC from rats sacrificed 30 minutes after ICV injection of a) olanzapine or b) AICAR, relative to control rats (DMSO). Calculations are based on results from 6 rats for each treatment group, run in duplicate. Representative images for the calculated difference were selected. Each lane (pACC, pAMPK and β-actin) always represents results on the same gel from the same rat. * P≤0.05 *vs.* vehicle. ** P≤0.01 *vs.* vehicle. *** P≤0.001 *vs.* vehicle.

**Figure 2:**
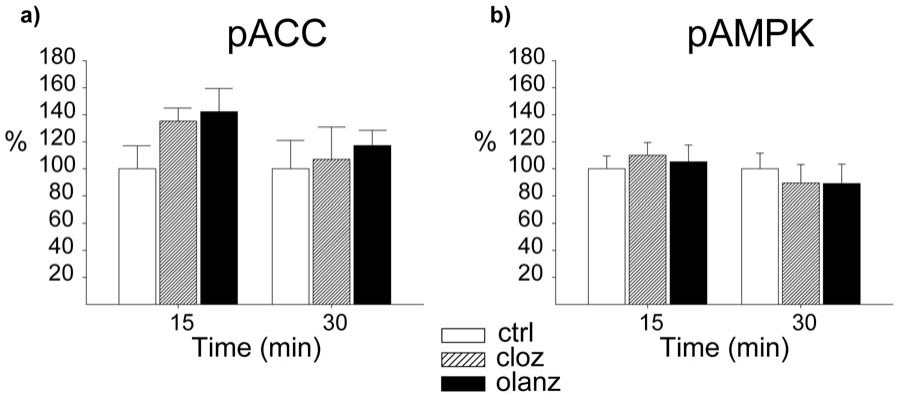
Effect of IP olanzapine and clozapine administration on phosphorylation of hypothalamic AMPK and ACC. Western blot analysis of hypothalamic levels of a) pACC or b) pAMPK in rats following IP injection of vehicle (ctrl) olanzapine (olanz; 10 mg/kg), clozapine (cloz; 25 mg/kg). Protein levels were normalized against β-actin as the endogenous control. Statistical calculations were based on results from n = 6 rats in each control group. * P≤0.05 *vs.* vehicle. ** P≤0.01 *vs.* vehicle. *** P≤0.001 *vs.* vehicle.

Intracerebroventricular (ICV) injection of olanzapine induced no clear sedative effects at doses up to 20 µg (evaluated through visual inspection), whereas a clear, but transient sedative effect was evident at 50 µg. We observed no effect on food intake, measured 1 h or 24 h after injection, at any of the doses tested (data not shown). It has been demonstrated that in an acute setting, antipsychotic agents induce hypothalamic activation of AMPK in rodents when administered at relatively high doses [22], [40]. We therefore measured the levels of phosphorylated (activated) AMPK (pAMPK) after ICV injection with 50 µg (the highest dose of olanzapine used in our experiment). No significant alteration in phosphorylation status was observed 30 minutes after the olanzapine injection relative to vehicle-treated controls, although we did see a trend towards increased levels of pAMPK (133±17%, P = 0.17) (Figure 1a). No significant effect was observed for phosphorylated acetyl-CoA carboxylase (pACC; 118±26%, P = 0.52), a downstream target of pAMPK (Figure 1a). In the same experimental setting, administration of the AMPK activator AICAR induced a significant increase of both pAMPK (170±17%, P<0.01) and pACC (295±54%, P<0.01) (Figure 1b). Similar data were obtained both for olanzapine and for AICAR 90 minutes after ICV injection (data not shown). Notably, antipsychotic-induced elevation of hypothalamic pAMPK levels has consistently been demonstrated in the acute setting after peripheral injection [22], [40]. We therefore performed an acute IP experiment, where we also included clozapine at a dose previously shown to induce marked metabolic changes in peripheral tissues [41]. In order to induce direct drug effects on AMPK phosphorylation, we chose to use relatively high doses of both olanzapine (10 mg/kg) and clozapine (25 mg/kg) in the IP experiment. It should be noted that sedative effects were evident (by visual inspection) for both drugs, precluding measurements of food intake. Neither clozapine nor olanzapine induced significant changes in the levels of pAMPK (Figure 2a) or pACC (Figure 2b), 15 and 30 minutes after injection. Still, a non-significant trend towards increased pACC levels was observed for both olanzapine (135±10%, P = 0.09) and clozapine (142±17%, P = 0.11) 15 minutes after injection (Figure 2a).

### Subchronic administration of olanzapine increases food intake and body weight

**Figure 3:**
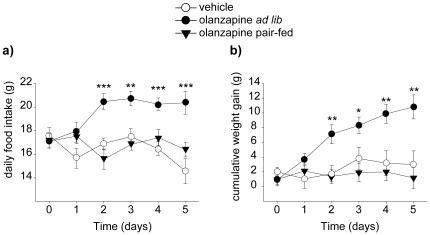
Food intake and body weight following subchronic administration of olanzapine. a) Daily average food intake in groups of rats (n = 8) exposed to olanzapine or vehicle by gavage (b.i.d) for 5 consecutive days. Rats were fasted over night and sacrificed in the morning on day 6. b) Cumulative weight gain in groups of rats (n = 8) treated with vehicle or olanzapine for 5 consecutive days. Total relative weight gain (mean±SEM), relative to treatment day 0 was as follows: control 3.0±1.8 g, olanzapine *ad libitum* 10.8±1.6 g, olanzapine pair-fed 1.2±1.4 g. * P≤0.05 *vs.* vehicle. ** P≤0.01 *vs.* vehicle. *** P≤0.001 *vs.* vehicle.

Next, we investigated the effect of subchronic olanzapine exposure (6 mg/kg/day) on food intake (Figure 3a) and weight gain (Figure 3b) in female rats. Repeated-measures two-way ANOVA was performed for daily food intake with treatment (2 groups) and time (6 days, including day 0, when starting the treatment) as factors. The analysis for six different time points revealed a significant main effect of the treatment [F(1,21) = 14.27; p<0.01] and a significant treatment x time interaction effect [F(5,17) = 5.24; p<0.01]. Each time point was subsequently analysed using Student's t-test (since only two treatment groups were present), revealing that daily food intake was significantly increased in the olanzapine *ad libitum* group from day 2 onwards (p<0.05). Similarly, cumulative body weight gain was analyzed using a two-way ANOVA repeated measures with treatment (3 groups) and time (6 days) as factors. Both significant main effect [F(2,45) = 8.15; p<0.01] and significant treatment x time interaction [F(10,82) = 2.52; p<0.05] effect were observed. Olanzapine-induced body weight gain was significantly increased from control from day 2 onwards (p<0.05) as determined from one-way ANOVA analysis, followed by Tukey's Post-hoc test. Any sedation caused by the moderately high dose of olanzapine used (6 mg/kg/day) may potentially have weight-promoting effects. Locomotor activity was measured only by visual inspection, which is a weakness in this study. Still, in pair-fed rats offered the same amount food as control rats, olanzapine exposure did not induce significant weight gain relative to vehicle-treated controls at any time point (Figure 3b). These data suggested that the weight-promoting effect of olanzapine is dependent on its orexigenic effects.

### Subchronic administration of olanzapine does not affect serum leptin, insulin, or adiponectin levels

Antipsychotic-induced weight gain has been suggested to be related to alterations in leptin, adiponectin and insulin serum levels [28], [42]. In our study, subchronic olanzapine exposure did not significantly alter serum levels of any of these endocrine factors, despite marked hyperphagia and weight gain (Table 1).

**Table 1:** Leptin, adiponectin and insulin plasma levels in control and olanzapine (*ad libitum* and pair-fed) treated rats.

	ctrl	olanz pair-fed	olanz *ad lib*
**Leptin (ng/ml)**	1.73±0.19	1.44±0.14	1.94±0.26
**Adiponectin (µg/ml)**	7.96±0.79	8.29±0.56	7.63±0.85
**Insulin (ng/ml)**	0.44±0.04	0.36±0.02	0.41±0.02

Data are expressed as mean±SEM.

### Subchronic olanzapine administration decreases hypothalamic AMPK phosphorylation

**Figure 4:**
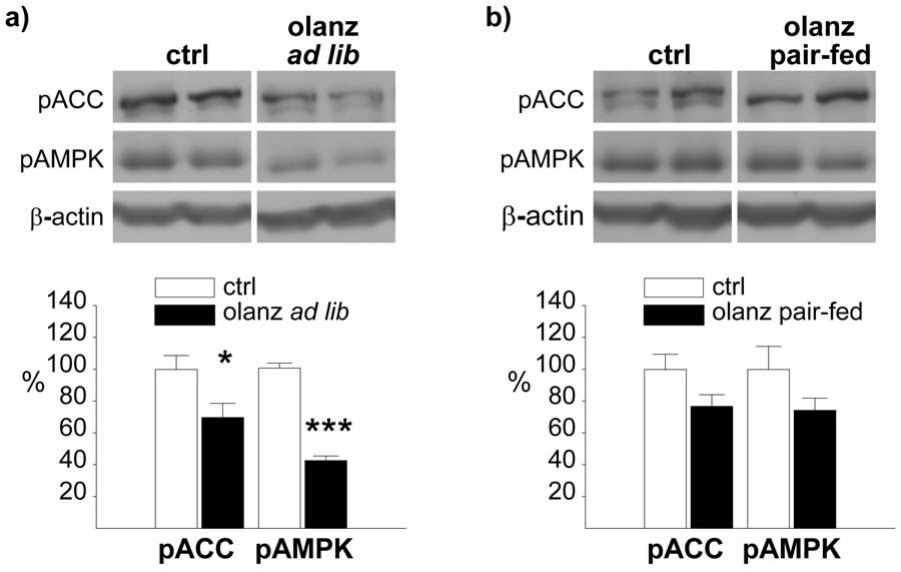
Effect of subchronic olanzapine administration on phosphorylation of hypothalamic AMPK and ACC. Western blot analysis of hypothalamic pAMPK and pACC from rats following an over night fast after 5 consecutive days of administration by gavage (b.i.d) with a) olanzapine (*ad libitum* fed) or b) olanzapine (pair-fed), relative to control rats. Calculations are based on results from 6 rats for each treatment group, run in duplicate. Representative images for the calculated difference were selected. Each lane (pACC, pAMPK and β-actin) always represents results on the same gel from the same rat. * P≤0.05 *vs.* vehicle. ** P≤0.01 *vs.* vehicle. *** P≤0.001 *vs.* vehicle.

In the subchronic setting, hypothalamic pAMPK levels were measured after 5 days of olanzapine exposure. Interestingly, we found that pAMPK levels in the hypothalamus of olanzapine-treated, *ad libitum*-fed rats were significantly reduced (42±3%, P<0.0001) relative to vehicle-treated controls (Figure 4a). Accordingly, olanzapine reduced the levels of phosphorylated acetyl-CoA carboxylase (pACC; 70±9%, P<0.05) in *ad libitum*-fed rats. No significant changes were observed in pair-fed rats, neither for pAMPK nor for pACC (Figure 4b).

### Subchronic olanzapine treatment increases mRNA expression of AgRP and NPY and decreases POMC in the ARC

**Figure 5:**
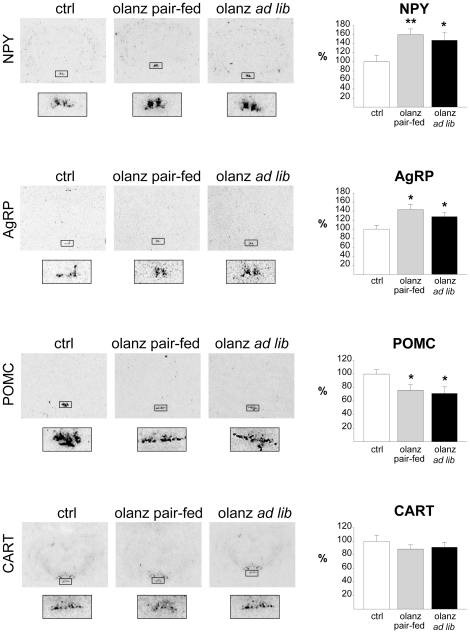
Effect of subchronic olanzapine administration on appetite-regulating neuropeptides. Expression levels of the appetite-regulating neuropeptides in the arcuate nucleus following 5 days of treatment (b.i.d) with vehicle (ctrl), olanzapine with food restriction (olanz pair-fed) or olanzapine with free access to food (olanz ad libitum). Calculations are based on results from groups of rats (n = 8) from each treatment group fasted over night and killed in the morning on day 6. Representative images demonstrating the calculated differences were selected. Delineated areas are shown at higher magnification at the bottom. * P≤0.05 *vs.* vehicle. ** P≤0.01 *vs.* vehicle. *** P≤0.001 *vs.* vehicle.

The observation that pAMPK levels were reduced in the subchronic experiment was inconsistent with a role of AMPK activation in olanzapine-induced hyperphagia. We therefore assayed the expression of key ARC neuropeptides involved in the control of food intake by using *in situ* hybridization analysis, considered the most suitable and robust approach for quantitative mRNA studies in the hypothalamus. In line with the elevated food intake observed in *ad libitum*-fed rats, olanzapine treatment increased mRNA levels of the orexigenic neuropeptides NPY (147±18%, P<0.05; Figure 5a) and AgRP (127±9%, P<0.05; Figure 5b) and reduced mRNA levels of the anorexigenic POMC (71±10%, P<0.05) in the ARC (Figure 5c) Similar results were observed in pair-fed rats that had not gained weight, with a marked increase in NPY (160±12%, P≤0.01) and AgRP (143±12%, P≤0.05) and reduced levels of POMC (76±8%, P≤0.05). Overall, these data suggest that the changes in neuropeptides do not represent secondary effects of olanzapine-induced hyperphagia. The expression level of the anorexigenic neuropeptide precursor CART did not change significantly in any of the olanzapine-treated groups (Figure 5d).

## Discussion

In this study, we investigated acute and subchronic effects of olanzapine exposure on hypothalamic AMPK as well as subchronic effects on satiety-regulating neuropeptides in female rats. In accordance with olanzapine-induced hyperphagia and increased body weight in the subchronic setting, we observed increased mRNA expression of the orexigenic neuropeptides NPY and AgRP, and decreased expression of the anorexigenic neuropeptide precursor POMC in the ARC. Interestingly, these changes were also observed in pair-fed rats, with restricted food intake and no weight gain, demonstrating that the olanzapine-induced transcriptional changes were primarily caused by antipsychotic treatment and did not occur secondary to alterations in feeding pattern and weight changes. Contrary to our initial hypothesis, we found that olanzapine reduced phosphorylated levels of AMPK, suggesting that hypothalamic AMPK activation is not the primary mechanism mediating olanzapine-induced neuropeptide expression and thus hyperphagia and weight gain in the subchronic setting. With respect to our acute experiments, no significant effect on AMPK phosphorylation status was observed.

It has been proposed that the molecular mechanisms underlying the appetite-stimulating effects of antipsychotic drugs may involve H1 receptor-mediated activation of hypothalamic AMPK [22]. This was supported by a recent study demonstrating AMPK activation in the hypothalamus of male rats following intravenous injection of olanzapine [40]. In our acute experiments, we observed a subtle trend towards increased levels of pAMPK after an acute ICV olanzapine injection and elevated pACC after an acute IP olanzapine injection. However, no accompanying measurements of food intake and body weight were reported in the aforementioned acute study [22]. In our acute study, the relatively high drug doses induced sedative effects, which potentially blunted hyperphagic effects.

Based on the recently established orexigenic effects of hypothalamic AMPK activation [15], [23], [25] and the previously suggested role of increased AMPK phosphorylation in antipsychotic-induced weight gain [22], it was somewhat unexpected that hypothalamic pAMPK levels and its molecular substrate pACC were reduced in our experimental setting. It is counterintuitive that AMPK does not mediate the hyperphagic and weight-promoting effects of olanzapine, and we speculate that AMPK phosphorylation may have been stimulated by olanzapine in the very short term (at initial time points) both in the acute and subchronic experiments, but that the elevation was not sustained at the time of dissection, around 20 hours after the last drug dose in subchronically treated rats. The reduction of pAMPK levels after subchronic olanzapine treatment was most pronounced in *ad libitum*-fed rats, which may suggest the involvement of negative feedback mechanisms triggered by increased body weight rather than a direct drug effect. Additionally, sedative effects may contribute to the weight gain observed in the hyperphagic olanzapine-treated *ad libitum* rats. However, the lack of significant weight gain in olanzapine-treated pair-fed rats strongly suggests that weight-inducing effect of sedation alone is unlikely.

The transcriptional changes of the appetite-regulating neuropeptides observed in our subchronic experiment are in accordance with a recent study in which acute ICV administration of olanzapine increased hypothalamic expression of both NPY and AgRP [40]. However, the expression of the anorexigenic POMC was not affected by olanzapine in this acute study, in contrast with our observation that POMC expression is reduced. In another subchronic study with an experimental design resembling ours, no effect was observed on the expression of hypothalamic neuropeptides after 7 days of olanzapine treatment in female rats, despite marked hyperphagia and weight gain [20]. These discrepancies are probably related to differences in experimental setup, including different drug doses (2 mg/kg/day versus 6 mg/kg/day in our study), the number of hours between the last drug dose and sacrifice, the duration of fasting before sacrifice, and particularly the use of real-time PCR analysis instead of the more sensitive *in situ* hybridization when assessing neuropeptide expression levels in specific neuronal populations. In this sense, in situ hybridization is a more suitable technique for studying neuropeptide expression, particularly relevant for neuropeptides expressed in more than one hypothalamic nucleus. This is the case of NPY, which is expressed both in the arcuate (ARC) and the dorsomedial nuclei (DMH), with ARC expression predominantly relevant in terms of feeding control [43], [44].

Additionally, our findings suggest that regulation of antipsychotic-induced appetite-controlling neuropeptides may occur without concomitant AMPK activation. This is supported by the aforementioned acute study by Martins et al. [40], demonstrating that hypothalamic AMPK activation by olanzapine occurs independently of food intake and without detectable neuropeptide expression changes following intravenous injection. Indeed, former studies have demonstrated that regulation of food intake and hypothalamic neuropeptides does not necessarily depend upon AMPK phosphorylation status. For instance, we previously showed that the anorectic effect of the drug tamoxifen is exerted by modulation of ARC neuropeptides through an AMPK-independent mechanism [24]. Also in line with our observations, recent findings have challenged the notion of a positive correlation between hyperphagia and AMPK activity, as demonstrated by reduced AMPK activation in hyperphagic, hyperthyroid rats [25] and by resistin-induced AMPK activation despite the anorexigenic effects of this hormone [45]. Furthermore, in the long-term setting, the orexigenic action of ghrelin is not mediated by increased AMPK activity and is also independent of neuropeptide tone [46], contrary to observations made in the acute setting [23], [25]. In fact, it was recently proposed both by us and by others that in long-term altered nutritional conditions, AMPK-induced changes in hypothalamic fatty acid metabolism may not play a key role in feeding control. In accordance with this hypothesis, it has been suggested that hypothalamic fatty acid metabolism could be a regulatory mechanism maintaining energy homeostasis in starvation [23], [47], [48], [49].

In summary, we show in this study that subchronic olanzapine exposure in female rats induces alterations in the expression of satiety-controlling neuropeptides in the ARC of hyperphagic rats, indicating that antipsychotic-induced weight gain may be mediated via changes in “classical” appetite-regulating neuropeptides. Of note, altered neuropeptide expression levels were also evident in food-restricted rats that did not gain weight, demonstrating that the olanzapine-induced changes are not secondary to changes in body weight and/or feeding patterns. In addition, we demonstrate that phosphorylation levels of AMPK are reduced by subchronic olanzapine exposure, suggesting that the role of AMPK in long-term antipsychotic-induced weight gain may be less robust than anticipated in previous acute studies. Overall, these data provide new insight into the hypothalamic mechanism underlying antipsychotic-induced hyperphagia and weight gain and provide a rationale for the search for alternative therapeutic targets to control energy balance.

## Materials and Methods

### Animals

All experiments were carried out in accordance with the guidelines of the Norwegian and Spanish Committees for Experiments on Animals. In accordance, experiments performed in Norway were approved by the Norwegian Committee for Experiments on Animals (Forsøksdyrutvalget, FDU), following standardized application through the animal facility at Haukeland University Hospital with ID 20092167. In the same way, all procedures performed in Spain were also approved by the University of Santiago de Compostela Institutional Bioethics Committee, the Xunta de Galicia (Local Government) and the Ministry of Science and Innovation with ID PS09/01880. Female, outbred Sprague-Dawley rats (Mollegaard, Denmark and the University of Santiago de Compostela Animal House) weighing between 230 g and 250 g on the first day of treatment were housed individually under standard conditions with an artificial 12:12 hrs light/dark cycle under constant 48% humidity. Animals were allowed free access to tap water and fed with standard laboratory chow during the experimental periods, as described below.

### Drugs

Olanzapine was dissolved in 0.1 M hydrochloric acid (HCl) and pH was adjusted to 5.5 using 0.1 M sodium hydroxide (NaOH). Stock solutions of 1.5 mg/ml were prepared and ∼0.5 ml of this solution was administered to the rats via gavage, twice daily (the actual volume was corrected for variation in body weight so that for each rat, each of the two daily doses was 3 mg/kg). For IP experiments, both olanzapine and clozapine solutions were prepared the same way, with the appropriate concentrations. For ICV injections, olanzapine and AICAR were dissolved in DMSO, which was used as vehicle.

### Acute experiments

Female rats had free access (*ad libitum*) to food and tap water throughout the experiment. Rats were acutely administered with olanzapine, either by intracerebroventricular (ICV) injection or by intraperitoneal (IP) injection. In the ICV experiment, cannulae were surgically implanted in rats as previously reported [23], [24], [25], [50]. After 3 days of recovery, rats were injected ICV with vehicle (DMSO, 10 µl), olanzapine (50 µg) or the AMPK activatior 5-aminoimidazole-4-carboxamide-1-d-ribofuranoside (AICAR; 50 µg) and sacrificed after 30 or 90 minutes. In the intraperitoneal (IP) experiment, female rats were sacrificed 15 or 30 minutes after administration of vehicle (saline), olanzapine (10 mg/kg) or clozapine (25 mg/kg). Whole brain was dissected out, frozen immediately on dry ice and stored at −80°C until processed.

### Subchronic experiment

Female rats were exposed to either vehicle (saline) or olanzapine (3 mg/kg), administered twice daily (total daily dose: 6 mg/kg) by gavage (9 a.m. and 3 p.m.) for 6 days, and sacrificed on day 7 after an overnight fast. The dose used is relatively high as compared to other studies, but has been shown to robustly induce hyperphagia and weight gain in mice [51] as well as in rats in our laboratory (unpublished results). In order to explore whether olanzapine could induce metabolic alterations independent of weight gain, we also included a pair-fed olanzapine-treated group in which the animals received an amount of food corresponding to that consumed by the control group during the previous 24 hours. To avoid binge eating, the pair-fed animals received 1/3 of the relevant amount of chow at 9.30 a.m., and the remaining 2/3 at 3 p.m. each day. Food intake and weight were measured daily for each animal. The last drug dose prior to sacrifice was administered 18–20 hours prior to decapitation. All animals were fasted from 9 p.m. on the day prior to euthanasia, with dissection starting at 9 a.m. the following day. Prior to decapitation, animals were anesthesized using isoflourane. Like in the acute experiment, whole brain was dissected out from all animals, frozen immediately on dry ice and stored at −80°C until processed. The brains were either used for *in situ* hybridization analysis (half of the animals) or western blot analysis (other half).

### Serum insulin, leptin and adiponectin measurements

Truncal vein blood was collected in EDTA tubes, left on ice for 30 minutes and centrifuged at 3,000 rpm for 10 minutes. Serum was transferred to pre-cooled Eppendorf tubes immediately after centrifugation and stored at -20°C. Serum insulin, leptin and adiponectin levels were assessed by means of a double-antibody radioimmunoassay (Linco Research, USA), as previously described [23], [25], [46]. All samples were assayed in duplicate within one assay, and the results were expressed in terms of the insulin, leptin or adiponectin standards.

### *In situ* hybridization

Coronal hypothalamic sections (16 µm) were cut on a cryostat and immediately stored at −80°C until hybridization. We used specific oligos for detection of AgRP, NPY, CART and POMC mRNAs. These probes were 3′-end labeled with 35S-αdATP using terminal deoxynucleotidyl transferase (Amersham Biosciences, UK). We performed *in situ* hybridizations as previously published [24], [25], [52]. Similar anatomical regions were analyzed using the rat brain atlas of Paxinos & Watson [53]. The slides from all experimental groups were exposed on the same autoradiographic film. All sections were scanned and the specific hybridization signal was quantified by densitometry using the ImageJ software (National Institute of Health, USA). We determined the optical density of the hybridization signal and subsequently corrected by the optical density of its adjacent background value. A rectangle, with the same dimensions in each case, was drawn enclosing the hybridization signal over each nucleus and over adjacent brain areas of each section (background) as previously described [24], [25], [52]. For the *in situ* analysis we included 8 animals per experimental group. We used between 16 and 20 sections for each animal (4–5 slides with four sections per slide). The mean of these 16–20 values was used as the densitometry value for each animal.

### Western blotting

Dissected hypothalami were homogenized in lysis buffer and centrifuged at 12000 g for 10 minutes at 4°C. 40 µg of total protein from each sample were separated on SDS-PAGE gels and blotted onto PVDF membranes. PVDF membranes were blocked with 5% BSA in 0.1% TBST prior to incubation with primary antibody at 4°C overnight, followed by incubation with secondary antibody at room temperature for one hour, as previously described [23], [52]. The primary antibodies used were: pACCα-Ser^79^ (Upstate, USA), pAMPKα-Thr^172^ (Cell signalling Technology, USA) and β-actin (Abcam, UK). Signal intensity measurements were performed using the ImageJ software (National Institutes of Health, USA).

### Statistical analysis

Food intake in the subchronic experiment was analyzed by two-way ANOVA repeated measures with treatment (2 groups; control and olanzapine ad libitum fed) as between-subject variable and time (6 days) as within-subject variable. Body weight changes was analyzed using the same method, with treatment (3 groups; control, olanzapine ad libitum fed and olanzapine pair-fed) and time (6 days) as factors. When a significant interaction effect from the two-way ANOVA was obtained, Student's t-test or one-way ANOVA, followed by Tukey's post-hoc test, was used to analyze statistical significance for each time point. All data are expressed as mean±SEM. All tests were conducted with PASW Statistics Version 18 (PASW statistics; SPSS, USA) software. A significance level of P = 0.05 was used.

## References

[1] Allison DB, Mentore JL, Heo M, Chandler LP, Cappelleri JC (1999). Antipsychotic-induced weight gain: a comprehensive research synthesis.. Am J Psychiatry.

[2] American Diabetes Association APAAmerican Association of Clinical EndocrinologistsNorth American Association for the Study of Obesity (2004). Consensus development conference on antipsychotic drugs and obesity and diabetes.. J Clin Psychiatry.

[3] Colton CW, Manderscheid RW (2006). Congruencies in increased mortality rates, years of potential life lost, and causes of death among public mental health clients in eight states.. Prev Chronic Dis.

[4] Weiden PJ, Mackell JA, McDonnell DD (2004). Obesity as a risk factor for antipsychotic noncompliance.. Schizophr Res.

[5] Blouin M, Tremblay A, Jalbert ME, Venables H, Bouchard RH (2008). Adiposity and eating behaviors in patients under second generation antipsychotics.. Obesity (Silver Spring).

[6] Cooper GD, Pickavance LC, Wilding JP, Halford JC, Goudie AJ (2005). A parametric analysis of olanzapine-induced weight gain in female rats.. Psychopharmacology (Berl).

[7] Pouzet B, Mow T, Kreilgaard M, Velschow S (2003). Chronic treatment with antipsychotics in rats as a model for antipsychotic-induced weight gain in human.. Pharmacol Biochem Behav.

[8] Hartfield AW, Moore NA, Clifton PG (2003). Effects of clozapine, olanzapine and haloperidol on the microstructure of ingestive behaviour in the rat.. Psychopharmacology (Berl).

[9] Hartfield AW, Moore NA, Clifton PG (2006). Effects of atypical antipsychotic drugs on intralipid intake and cocaine-induced hyperactivity in rats.. Neuropsychopharmacology.

[10] Nasrallah HA (2008). Atypical antipsychotic-induced metabolic side effects: insights from receptor-binding profiles.. Mol Psychiatry.

[11] Reynolds GP, Kirk SL (2010). Metabolic side effects of antipsychotic drug treatment--pharmacological mechanisms.. Pharmacol Ther.

[12] Carling D (2004). The AMP-activated protein kinase cascade--a unifying system for energy control.. Trends Biochem Sci.

[13] Hu Z, Cha SH, Chohnan S, Lane MD (2003). Hypothalamic malonyl-CoA as a mediator of feeding behavior.. Proc Natl Acad Sci U S A.

[14] Kahn BB, Alquier T, Carling D, Hardie DG (2005). AMP-activated protein kinase: ancient energy gauge provides clues to modern understanding of metabolism.. Cell Metab.

[15] Lage R, Diéguez C, Vidal-Puig A, López M (2008). AMPK: a metabolic gauge regulating whole-body energy homeostasis.. Trends Mol Med.

[16] López M, Lelliott CJ, Vidal-Puig A (2007). Hypothalamic fatty acid metabolism: a housekeeping pathway that regulates food intake.. Bioessays.

[17] Morton GJ, Cummings DE, Baskin DG, Barsh GS, Schwartz MW (2006). Central nervous system control of food intake and body weight.. Nature.

[18] Kirk SL, Cahir M, Reynolds GP (2006). Clozapine, but not haloperidol, increases neuropeptide Y neuronal expression in the rat hypothalamus.. J Psychopharmacol.

[19] Huang XF, Deng C, Zavitsanou K (2006). Neuropeptide Y mRNA expression levels following chronic olanzapine, clozapine and haloperidol administration in rats.. Neuropeptides.

[20] Davoodi N, Kalinichev M, Korneev SA, Clifton PG (2009). Hyperphagia and increased meal size are responsible for weight gain in rats treated sub-chronically with olanzapine.. Psychopharmacology (Berl).

[21] Guesdon B, Denis RG, Richard D (2010). Additive effects of olanzapine and melanin-concentrating hormone agonism on energy balance.. Behav Brain Res.

[22] Kim SF, Huang AS, Snowman AM, Teuscher C, Snyder SH (2007). From the Cover: Antipsychotic drug-induced weight gain mediated by histamine H1 receptor-linked activation of hypothalamic AMP-kinase.. Proc Natl Acad Sci U S A.

[23] López M, Lage R, Saha AK, Pérez-Tilve D, Vázquez MJ (2008). Hypothalamic fatty acid metabolism mediates the orexigenic action of ghrelin.. Cell Metab.

[24] López M, Lelliott CJ, Tovar S, Kimber W, Gallego R (2006). Tamoxifen-induced anorexia is associated with fatty acid synthase inhibition in the ventromedial nucleus of the hypothalamus and accumulation of malonyl-CoA.. Diabetes.

[25] López M, Varela L, Vázquez MJ, Rodriguez-Cuenca S, Gonzalez CR (2010). Hypothalamic AMPK and fatty acid metabolism mediate thyroid regulation of energy balance.. Nat Med.

[26] Minokoshi Y, Alquier T, Furukawa N, Kim YB, Lee A (2004). AMP-kinase regulates food intake by responding to hormonal and nutrient signals in the hypothalamus.. Nature.

[27] Lane MD, Wolfgang M, Cha SH, Dai Y (2008). Regulation of food intake and energy expenditure by hypothalamic malonyl-CoA.. Int J Obes (Lond).

[28] Boyda HN, Tse L, Procyshyn RM, Honer WG, Barr AM (2010). Preclinical models of antipsychotic drug-induced metabolic side effects.. Trends Pharmacol Sci.

[29] Albaugh VL, Henry CR, Bello NT, Hajnal A, Lynch SL (2006). Hormonal and metabolic effects of olanzapine and clozapine related to body weight in rodents.. Obesity (Silver Spring).

[30] Cooper GD, Harrold JA, Halford JC, Goudie AJ (2008). Chronic clozapine treatment in female rats does not induce weight gain or metabolic abnormalities but enhances adiposity: implications for animal models of antipsychotic-induced weight gain.. Prog Neuropsychopharmacol Biol Psychiatry.

[31] Goudie AJ, Smith JA, Halford JC (2002). Characterization of olanzapine-induced weight gain in rats.. J Psychopharmacol.

[32] Arjona AA, Zhang SX, Adamson B, Wurtman RJ (2004). An animal model of antipsychotic-induced weight gain.. Behav Brain Res.

[33] Coccurello R, Brina D, Caprioli A, Conti R, Ghirardi O (2009). 30 days of continuous olanzapine infusion determines energy imbalance, glucose intolerance, insulin resistance, and dyslipidemia in mice.. J Clin Psychopharmacol.

[34] Coccurello R, Caprioli A, Conti R, Ghirardi O, Borsini F (2008). Olanzapine (LY170053, 2-methyl-4-(4-methyl-1-piperazinyl)-10H-thieno[2,3-b][1,5] benzodiazepine), but not the novel atypical antipsychotic ST2472 (9-piperazin-1-ylpyrrolo[2,1-b][1,3]benzothiazepine), chronic administration induces weight gain, hyperphagia, and metabolic dysregulation in mice.. J Pharmacol Exp Ther.

[35] Coccurello R, Caprioli A, Ghirardi O, Conti R, Ciani B (2006). Chronic administration of olanzapine induces metabolic and food intake alterations: a mouse model of the atypical antipsychotic-associated adverse effects.. Psychopharmacology (Berl).

[36] Coccurello R, D'Amato FR, Moles A (2008). Chronic administration of olanzapine affects Behavioral Satiety Sequence and feeding behavior in female mice.. Eat Weight Disord.

[37] Fell MJ, Marshall KM, Williams J, Neill JC (2004). Effects of the atypical antipsychotic olanzapine on reproductive function and weight gain in female rats.. J Psychopharmacol.

[38] Minet-Ringuet J, Even PC, Goubern M, Tome D, de Beaurepaire R (2006). Long term treatment with olanzapine mixed with the food in male rats induces body fat deposition with no increase in body weight and no thermogenic alteration.. Appetite.

[39] Minet-Ringuet J, Even PC, Lacroix M, Tome D, de Beaurepaire R (2006). A model for antipsychotic-induced obesity in the male rat.. Psychopharmacology (Berl).

[40] Martins PJ, Haas M, Obici S (2010). Central nervous system delivery of the antipsychotic olanzapine induces hepatic insulin resistance.. Diabetes.

[41] Ferno J, Vik-Mo AO, Jassim G, Havik B, Berge K (2009). Acute clozapine exposure in vivo induces lipid accumulation and marked sequential changes in the expression of SREBP, PPAR, and LXR target genes in rat liver.. Psychopharmacology (Berl).

[42] Jin H, Meyer JM, Mudaliar S, Jeste DV (2008). Impact of atypical antipsychotic therapy on leptin, ghrelin, and adiponectin.. Schizophr Res.

[43] López M, Seoane LM, Tovar S, García MC, Nogueiras R (2005). A possible role of neuropeptide Y, agouti-related protein and leptin receptor isoforms in hypothalamic programming by perinatal feeding in the rat.. Diabetologia.

[44] López M, Tovar S, Vázquez MJ, Williams LM, Diéguez C (2007). Peripheral tissue-brain interactions in the regulation of food intake.. Proc Nutr Soc.

[45] Vazquez MJ, Gonzalez CR, Varela L, Lage R, Tovar S (2008). Central resistin regulates hypothalamic and peripheral lipid metabolism in a nutritional-dependent fashion.. Endocrinology.

[46] Sangiao-Alvarellos S, Varela L, Vazquez MJ, Da Boit K, Saha AK (2010). Influence of ghrelin and growth hormone deficiency on AMP-activated protein kinase and hypothalamic lipid metabolism.. Journal of neuroendocrinology.

[47] Andrews ZB, Liu ZW, Walllingford N, Erion DM, Borok E (2008). UCP2 mediates ghrelin's action on NPY/AgRP neurons by lowering free radicals.. Nature.

[48] Varela L, Vazquez MJ, Cordido F, Nogueiras R, Vidal-Puig A (2011). Ghrelin and lipid metabolism: key partners in energy balance.. Journal of molecular endocrinology.

[49] López M (2008). The AMPK-malonyl-CoA-CPT1 axis in the control of hypothalamic neuronal function.. Cell Metab.

[50] Lage R, Vazquez MJ, Varela L, Saha AK, Vidal-Puig A (2010). Ghrelin effects on neuropeptides in the rat hypothalamus depend on fatty acid metabolism actions on BSX but not on gender.. The FASEB journal : official publication of the Federation of American Societies for Experimental Biology.

[51] Cope MB, Nagy TR, Fernandez JR, Geary N, Casey DE (2005). Antipsychotic drug-induced weight gain: development of an animal model.. Int J Obes (Lond).

[52] Chakravarthy MV, Zhu Y, López M, Yin L, Wozniak DF (2007). Brain fatty acid synthase activates PPARalpha to maintain energy homeostasis.. The Journal of clinical investigation.

[53] Paxinos G (1986). The rat brain in stereotaxic coordinates..

